# Integrative Role of RNA N7-methylguanosine in epilepsy: Regulation of neuronal oxidative phosphorylation, programmed death and immune microenvironment

**DOI:** 10.1371/journal.pone.0327256

**Published:** 2025-07-14

**Authors:** Jiangli Zhao, Qingyuan Sun, Xuchen Liu, Jiwei Wang, Ning Yang, Chao Li, Xinyu Wang

**Affiliations:** 1 Department of Neurosurgery, Qilu Hospital, Cheeloo College of Medicine and Institute of Brain and Brain-Inspired Science, Shandong University, Jinan, China; 2 Jinan Microecological Biomedicine Shandong Laboratory, Jinan, China; 3 Shandong Key Laboratory of Brain Function Remodeling, Jinan, China; 4 School of Medicine, Cheeloo College of Medicine, Shandong University, Jinan, China; Alfaisal University, SAUDI ARABIA

## Abstract

Epilepsy is a common brain disease that causes different types of seizures, with an incidence rate of nearly 1%. N7-methylguanosine (m7G) is a prevalent RNA modification that has attracted significant attention in recent research. In this study, we investigated the regulatory pattern and clinical significance of m7G methylation in epilepsy. Gene expression analysis of datasets GSE143272 and GSE190452 identified 8 differentially expressed m7G regulators (NUDT3, EIF4E3, LARP1, IFIT5, SNUPN, METTL1, EIF4A1, and LSM1) in epilepsy. Through consensus clustering, epilepsy patients were divided into two molecular subtypes based on m7G patterns. Enrichment and immune infiltration analyses revealed differences in immune cell infiltration and functions between the two subtypes, particularly in the levels of CD8^+^ T cells and cytolytic activity. Our findings also suggested that active m7G levels could promote oxidative phosphorylation in the neurons of epilepsy patients and decrease neuronal necroptosis activity. Machine learning algorithms were used to identify key m7G regulators (EIF4E3, NUDT3, SNUPN, LSM1, and METTL1), and a nomogram model was constructed based on these findings. Validation with serums and tissue samples from healthy controls and epilepsy patients confirmed the RNA expression levels of the identified m7G regulators. Overall, this study highlights the important role of m7G regulators in the immune microenvironment, cellular death, and oxidative phosphorylation in epilepsy patients. The insights gained from this research could potentially guide future therapy strategies for epilepsy patients and improve their outcomes.

## Introduction

Epilepsy is a prevalent serious neurologic disorder that can be challenging to fully cure, as approximately 30% of patients do not respond to medication [1]. According to the WHO 2010 Global Burden of Disease study, epilepsy is ranked as the second most burdensome neurologic disorder worldwide in terms of disability-adjusted life years [2]. The causes of epilepsy can be classified into different categories including structural, genetic, infectious, metabolic, immune, and other factors [3]. The choice of the appropriate treatment approach is critical for these complicated causes. Targeted medication repurposing based on single gene abnormalities has demonstrated potential in treating epilepsy caused by mutations that cause gain-of-function in ion-channel subunit genes [4]. To create stable and efficient antiepileptic medications, however, utilizing certain genes and their products as intervention sites, requires a present lack of information.

N7-methylguanosine (m7G) modification is one of the most prevalent RNA modifications [5] which often located at the 5’ caps and internal positions of eukaryotic mRNA or internally within rRNA and tRNA of all species [6,7], and enriched at the 5’UTR region and AG-rich contexts [8]. It is crucial for controlling splicing, translation, and mRNA export, which in turn controls mRNA translation, self-renewal, pluripotency, and neural lineage differentiation [9]. In cancer therapy and drug resistance, the roles of RNA-modifying proteins and RNA modifications like m7G and N6-methyladenosine (m6A) are regularly investigated [10,11]. The modifications have profound significance in the development and growth of animals and humans [12,13], while abnormal myelin sheath development is regarded as an important cause of epilepsy. We have previously established a significant correlation between m6A and epilepsy [14]. Consequently, we have solid grounds for thinking that m7G could likewise be the pertinent epilepsy mechanism.

In order to assess the activities of m7G associated genes and their involvement in epilepsy, the Gene Expression Omnibus (GEO) GSE143272 dataset, which investigated the human peripheral whole blood sample microarray dataset, was studied. Using single-cell sequencing data of GSE190452’s epileptic brain tissue, we investigated the connection between m7G and epilepsy in further detail at the single-cell level. We separated m7G patients into two groups based on m7G regulators that had changed expression in a subset of epilepsy patients. Simultaneously, we screened the important m7G regulators in epilepsy using several machine learning techniques, and based on those results, we constructed a nomogram model to predict the risk of epilepsy. Our findings illustrated the critical function that m7G plays in epilepsy and offered fresh perspectives for future studies on epigenetics and epilepsy therapy in the clinic.

## Results

### Identification of significant m7G regulators in epilepsy

**Fig 1:**
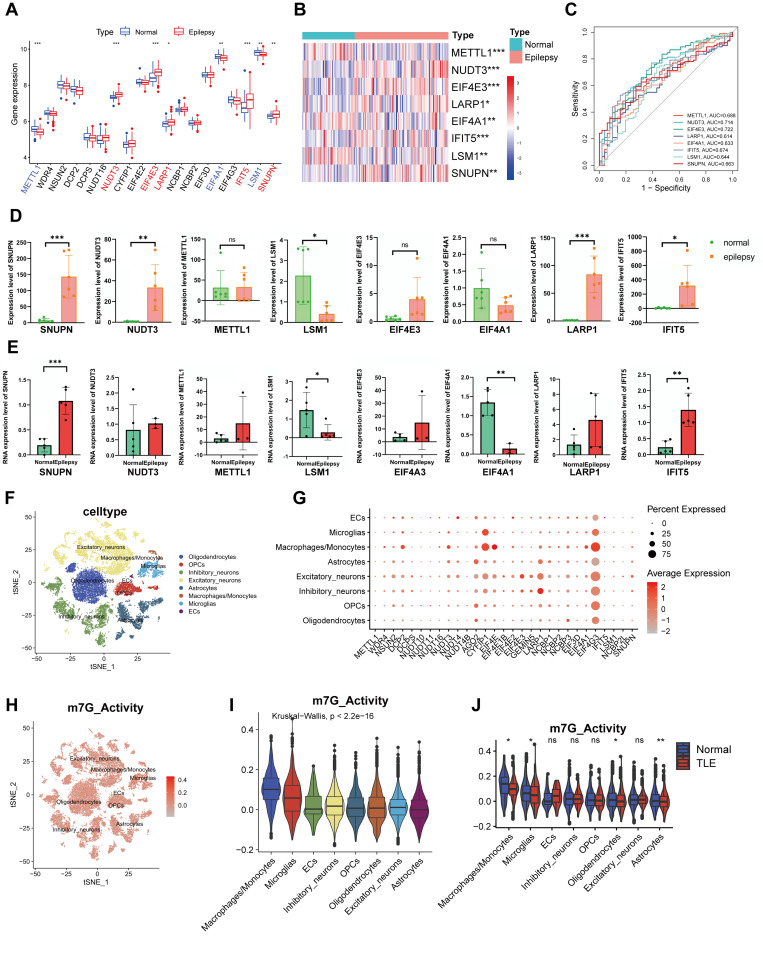
Expression of RNA G7-methyladenosine (m7G) regulators in epilepsy. (A) Expression landscape of m7G regulators in normal and epilepsy persons. (B) Heat map of differentially expressed m7G regulators. (C) ROC curve of 8 significant m7G regulators. Validation of expression of 8 significant m7G regulators in epilepsy by RT-qPCR: blood tissue (D) and brain tissue (E). (F) TSNE plot of brain cells from 4 normal and 4 epilepsy persons. (G) Expression pattern of m7G regulators in the different cell types. (H) Feature plot of m7G activity in each cell. (I) Difference of m7G activity among each cell type. (J) Down-regulation of m7G activity in a variety of cell types of patients with TLE. *p < 0.05, **p < 0.01, ***p < 0.001.

Eight of the 19 m7G regulators with expression data that were retrieved from GSE143272 were found to be important m7G regulators. The boxplot revealed that epilepsy patients had up-regulated expression levels of NUDT3, EIF4E3, LARP1, IFIT5, and SNUPN, and down-regulated expression levels of METTL1, EIF4A1, and LSM1 (Fig 1A). Significant m7G gene expression was displayed on a heat map (Fig 1B). With an AUC > 0.60, the ROC curve demonstrated their ability to differentiate between individuals with epilepsy and those without (Fig 1C). The sensitivity and specificity for each differentially expressed gene are presented in Table 1. The expression levels of major m7G genes in the whole blood (Fig 1D) and brain tissue (Fig 1E) and of patients with epilepsy and non-epileptic disorders were compared in detail based on the outcomes of real-time qPCR (RT-qPCR). Patients with epilepsy were more likely to exhibit aberrant expression of SNUPN, LSM1, and IFIT5.

**Table 1:** The sensitivity and specificity of each m7G differentially expressed regulators in epilepsy.

m7G genes	Cut-off	Specificity	Sensitivity
METTL1	5.42	0.55	0.78
NUDT3	7.36	0.61	0.77
ETF4E3	8.41	0.53	0.84
LARP1	5.97	0.76	0.48
EIF4A1	9.53	0.59	0.65
IFIT5	7.15	0.84	0.53
LSM1	9.80	0.74	0.57
SNUPN	6.35	0.73	0.59

m7G: N7-methylguanosine.

### Single cell sequencing showing an overview of m7G in temporal lobe epilepsy (TLE)

We were able to gather a general picture of the overall and specific gene expression levels of m7G-related genes in TLE by mining the dataset GSE190452. First, we determined the association between the nFeature RNA, nCount RNA, and the percentage of mitochondrial gene in each cell (S1A and 1B Figs). Batchability between individual samples is addressed following the normalization based on the “harmony” package (S1C and S1D Figs). Based on conventional markers, the tSNE map revealed that 22343 cells from eight different people were categorized into 36 groups and identified as eight distinct cell types (S1E, S1F Figs and Fig 1F). It is evident that there was variation in the expression levels of m7G-related genes in brain tissues, however EIF4G3 was highly expressed in the majority of cell types (Fig 1G). The m7G pathway’s activity in each cell was then determined, and the results showed that the mononuclear phagocyte system—which includes monocytes, macrophages, and microglia cells—had greater m7G activity (Figs 1H and 1I). Simultaneously, m7G activity was considerably downregulated in oligodendrocytes, astrocytes, and the mononuclear phagocyte system in TLE patients than control (Fig 1J)

### Identification of distinct m7G patterns by consensus clustering

**Fig 2:**
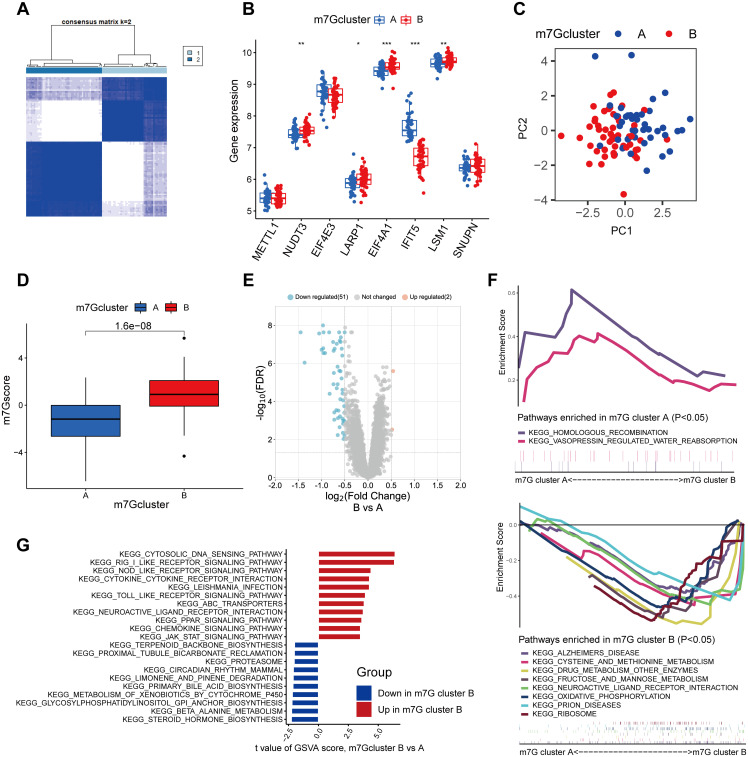
Consensus clustering of epilepsy patients based on significant m7G regulators. (A) Consensus matrix of patients when k = 2. (B) Differentially expression of 8 significant m7G regulators between m7G clusters. (C) Principal component analysis (PCA) based on significant m7G regulators. (D) Boxplot of m7G score of patients between m7G clusters. (E) Volcano map of DEGs, |logFC| > 0.5, fdr < 0.05 as cutoff criteria. (F) GSEA shows the KEGG pathways enriched in m7G cluster A or B. (G) Result of GSVA enrichment analysis between m7G cluster A and B. *p < 0.05, **p < 0.01, ***p < 0.001.

Eight major m7G regulators allowed us to separate the 91 epilepsy patients into two categories. We discovered that the grouping benefited most by consensus clustering when k = 2. (Fig 2A). Next, patients were separated into clusters A and B of m7G. Boxplot analysis and visualization of the expression of eight important m7G regulators in m7G clusters were performed. While IFIT5 was up-regulated in m7G cluster A, NUDT3, LARP1, EIF4A1, and LSM1 expression levels were up-regulated in m7G cluster B (Fig 2B). Furthermore, we used PCA to demonstrate how effectively this technique could separate patients from clusters (Fig 2C). While scores in m7G cluster A were typically lower than cluster B, the m7G scores of epilepsy from two different m7G patterns varied in m7G score (Fig 2D). After that, we conducted an analysis and identified the differentially expressed genes (DEGs) across the two m7G clusters of epileptic patients. In m7G cluster B patients, these genes included 51 genes with decreased expression and 2 genes with greater expression (Fig 2E). Patients with m7G cluster B epilepsy, or those with greater levels of m7G activity, had higher levels of oxidative phosphorylation, according to the GSEA (Fig 2F). Furthermore, the GSVA result revealed that some immune pathways, such as the chemokine signaling pathway, were also more activated in m7G cluster B in addition to innate immune pathways including RIG I like receptor signaling, NOD like receptor signaling, and TOLL like receptor signaling (Fig 2G). In order to validate our findings, we divided individuals with epilepsy into gene Cluster A and B using 53 DEGs, and then we compared the m7G scores of the two groups (S2A and S2B Figs). A heatmap representing the DEG expression levels in the two groups was shown (S2C Fig). There was a difference in the expression of three out of the eight important m7G genes between the two groups (S2D Figs). To put it briefly, there was a strong correlation between the outcomes of the first and second clustering.

### Estimation of immune microenvironment and functions

**Fig 3:**
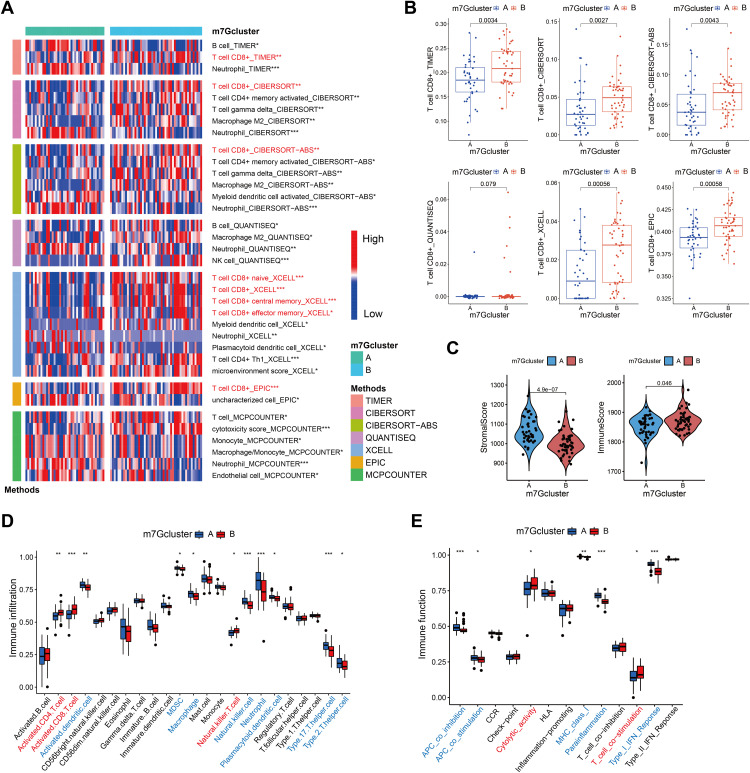
Analyses of Immune microenvironment and functions between m7G clusters. (A) Multi-algorithms suggest different infiltrated cell types between m7G clusters. (B) Multi-algorithms suggest that CD8+ T cells were more abundant in m7G cluster B. (C) Differences of stromal and immune scores between m7G clusters. (D-E) Differences of immune cell infiltration (D) and immune functions (E) between m7G clusters calculated by ssGSEA. *p < 0.05, **p < 0.01, ***p < 0.001.

We performed a very thorough investigation of the immune microenvironment in epilepsy patients since the earlier research suggested a relationship between m7G and immune-related pathways. Several immune microenvironment calculation techniques indicated that m7G cluster B included a larger concentration of CD8^+^ T cells (Fig 3A). Fig 3B displayed a boxplot with extra information. Furthermore, m7G cluster B epilepsy patients exhibited more immune cells overall but less stromal cells in their brain tissue, according to the ESTIMATE algorithm (Fig 3C). Additionally, eight important m7G genes showed high correlations with a range of immune cells based on the ssGSEA. For instance, there was a substantial correlation (spearman R = 0.46) between the quantity of activated CD8^+^T cells and LSM1 expression (S3A Fig). Concurrently, there was a notable rise in NKT cells and activated CD8^+^ T cells in m7G cluster B and gene cluster B (Fig 3D, S3B). Consequently, it is easy to see why these individuals’ cytolytic activity has increased (Fig 3E, S3C). To sum up, m7G activity in epileptic patients is strongly associated with their local immune microenvironment, primarily shown in its facilitation of CD8^+^ T cell infiltration and cytolytic potential.

### Identification of key m7G regulators through multiple machine learning algorithms

**Fig 4:**
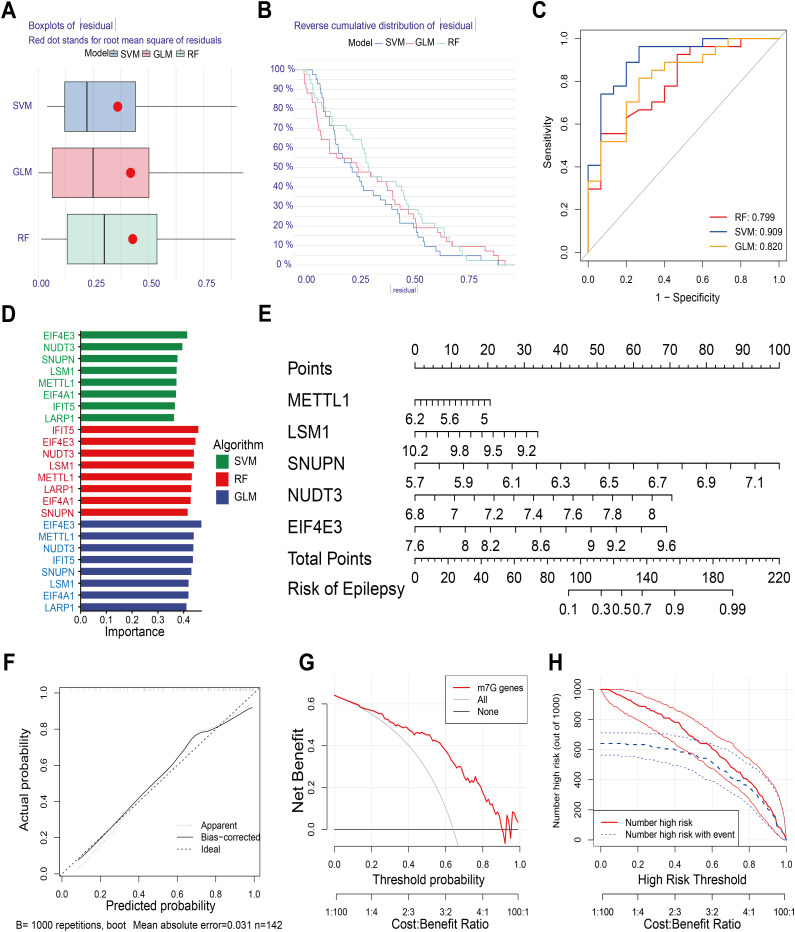
Construction of nomogram model through SVM algorithm. (A) Boxplot of |residual| of three algorithms: SVM, GLM, and RF. (B) Reverse cumulative distribution of |residual| of three algorithms. (C) ROC curve indicating the accuracy of three algorithms. (D) Importance of significant m7G regulators based on three algorithms. (E) Nomogram model using top5 most important significant m7G regulators through SVM algorithm. (F) Calibration curve of nomogram model. (G) DCA curve showing net benefit of nomogram model. (H) Analysis of the clinical impact of nomogram model.

We used three machine learning techniques (SVM, GLM, and RF) to investigate the relative significance and rank of eight important m7G regulators in the incidence of epilepsy. Each model’s |residual| was displayed in a boxplot, with SVM having the lowest residual (Fig 4A). On the other hand, Fig 4B shows that their reverse cumulative distribution of |residual| was almost equal. Additionally, based on the ROC curves, SVM was selected for the subsequent studies as it had the maximum AUC (Fig 4C). The relative significance of important m7G regulators was shown by the bar plot. Key m7G regulators were found to be EIF4E3, NUDT3, SNUPN, LSM1, and METTL1, which were listed as rankings in accordance with the SVM model (Fig 4D).

### Construction of nomogram model on the basis of key m7G regulators

To forecast the incidence of epilepsy, a nomogram model was constructed using five important m7G regulators (Fig 4E). With 1000 trials and a mean absolute error of 0.031, the calibration curve showed consistency between the expected value and the actual value, indicating that this model could accurately predict the risk of epilepsy (Fig 4F). Additionally, the DCA curve (Fig 4G) suggests that individuals with epilepsy may benefit from the nomogram model. Its exceptional predictive power was demonstrated by the clinical curve (Fig 4H).

### Relationship between m7G regulators and clinical factors

To investigate the relationship between m7G and clinical variables, we separated patients into multiple groups and examined the variations in their m7G scores. The heatmap showed the expression of 8 m7G important regulators in addition to clinical variables such as age, gender, type of epilepsy, drug-using, and drug-response in different m7G clusters. Additionally, two types of clustering data were included (S4A Fig). The baseline table showed the detailed clinical variables between distinct m7G clusters (S1 Table). We examined the variations in m7G scores within and between these clinical factor-based subgroups in depth. It is evident that there was no significant difference in m7G scores across groups based on age, gender, type, and drug-response (S4B–E Figs). This indicated that m7G is conservative and stable in epilepsy patients, and that additional non-traditional approaches were required to bring about this modification. Three grouping approaches that were strongly connected to each other were included in the Sankey diagram: the m7G cluster, gene cluster, and m7G score (S4F Fig).

### Regulation of oxidative phosphorylation by m7G in neurons of TLE patients

**Fig 5:**
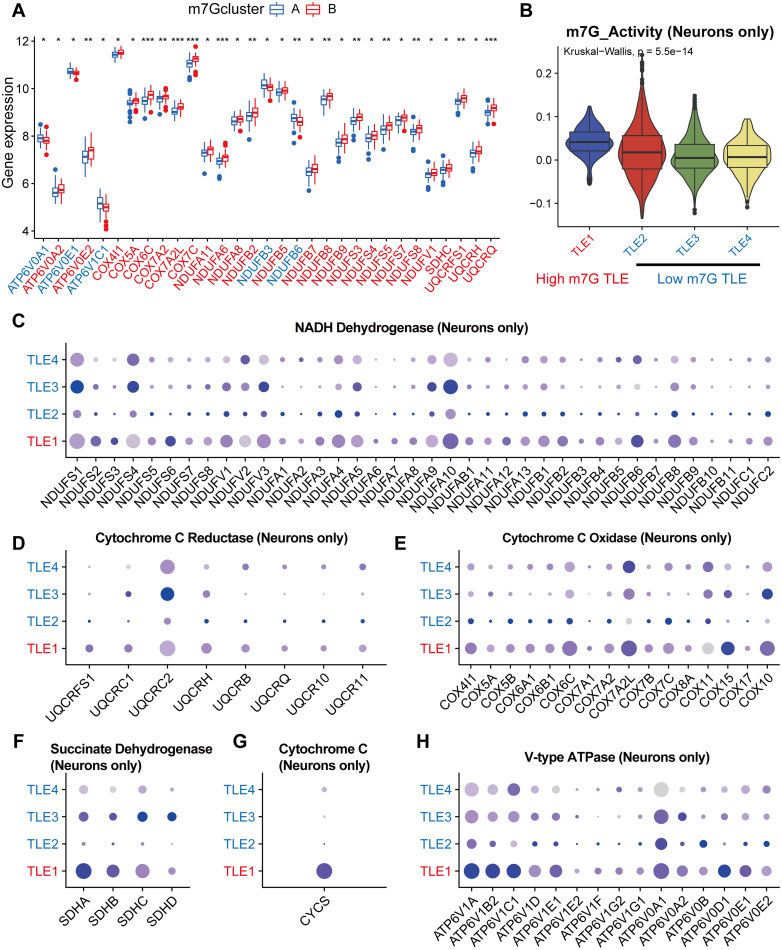
M7G regulators may improve oxidative phosphorylation of neurons in brain of epilepsy. (A) Differentially expressed genes related to oxidative phosphorylation between m7G clusters. (B) M7G scores of TLE patients’ neurons, TLE1 with higher scores than others. (C-H) Expression of oxidative phosphorylation related genes in each TLE patients’ neurons, including 6 parts, which are NADH Dehydrogenase (C), Cytochrome C Reductase (D), Cytochrome C Oxidase (E), Succinate Dehydrogenase (F), Cytochrome C (G), and V-type ATPase (H). *p < 0.05, **p < 0.01, ***p < 0.001.

Having established the general function of m7G in stimulating oxidative phosphorylation, we proceeded to investigate it in further detail. The boxplots demonstrate that the m7G cluster and gene cluster exhibit substantially divergent expression levels of genes associated to oxidative phosphorylation (Fig 5A, S5A Fig). Red indicates up-regulation and blue indicates down-regulation in cluster B on the X-axis. Furthermore, we computed each cell’s m7G activity using the “AddModuleScore” function, and we compared the m7G activity of all cells and neurons-only in four TLE patients, respectively. According to Figs 5B and S5B, TLE1 was classified as a high m7G TLE patient and the other three as low m7G TLE patients. The oxidative phosphorylation-related genes in the dataset were then roughly categorized into six groups based on KEGG’s resolution of the oxidative phosphorylation pathway. These groups included NADH Dehydrogenase, Cytochrome C Reductase, Cytochrome C Oxidase, Succinate Dehydrogenase, Cytochrome C, and V-type ATPase. First, it was evident that brain cells as a whole had increased expression of all six sets of genes in high m7G TLE (S5C–H Figs). The results were mostly consistent with just neurons, and in particular, the TLE1 patient had monopolistically expressed CYCS, the gene for cytochrome C (Figs 5C-H). In summary, our findings demonstrated a strong positive correlation between the oxidative phosphorylation activity and m7G activity in the brain neurons of TLE patients.

### Relationship between neuronal death and m7G activity

**Fig 6:**
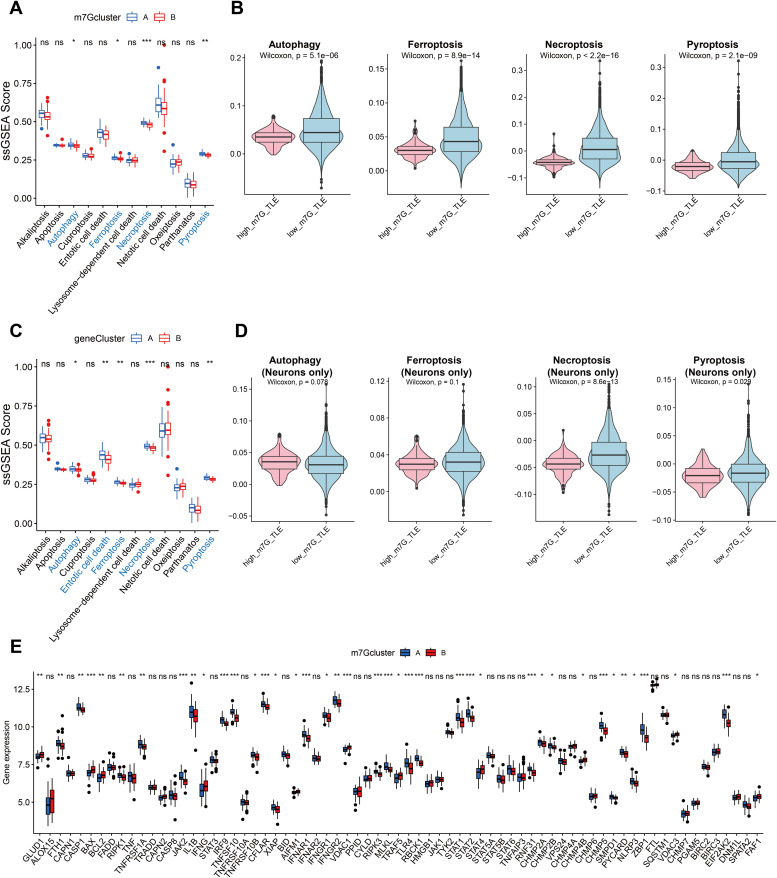
M7G could inhibit necroptosis in neurons of epilepsy patients. Differences of programmed cell death mode between m7G clusters (A) and gene clusters (C). Autophagy, ferroptosis, necroptosis, and pyroptosis is negatively correlated with m7G level in brain cells overall (B), and neurons-only (D). Detailed expression of necroptosis-related genes between m7G clusters (E). *p < 0.05, **p < 0.01, ***p < 0.001.

Research has indicated that seizures have the potential to cause neuronal death, and that neuronal death and acquired epileptogenesis are closely related [15]. As a result, we investigated the possible correlation between m7G and a total of 12 ways of cell death in further detail. The findings demonstrated that m7G and gene cluster B (the group with higher m7G scores) had decreased levels of autophagy, ferroptosis, necroptosis, and pyroptosis (Figs 6A and 6C). We also performed further validation with the single cell sequencing dataset of TLE at the same time. The findings of determining each cell’s m7G activity revealed that, in addition to the four cell death modes mentioned above, patients with greater m7G TLE also exhibited a majority down-regulation of the remaining eight cell death mechanisms (Figs 6B, S6A–H Figs). In order to investigate further, we limited our comparison to m7G scores in neurons. The findings indicated that necroptosis was the form of cell death that varied the most between the two groups (Figs 6D). As a result, we examined the variations in necroptosis-related gene expression across m7G clusters A and B. We discovered that most genes, including the initiator gene RIPK3 and the important intermediary gene MLKL, were expressed less in m7G cluster B (Fig 6E) [16]. In summary, our findings imply that increased m7G expression in TLE patients’ neurons appears to support neuronal survival, mainly by preventing necroptosis.

### Increased IFIT5 expression is causally related to the occurrence of epilepsy

**Fig 7:**
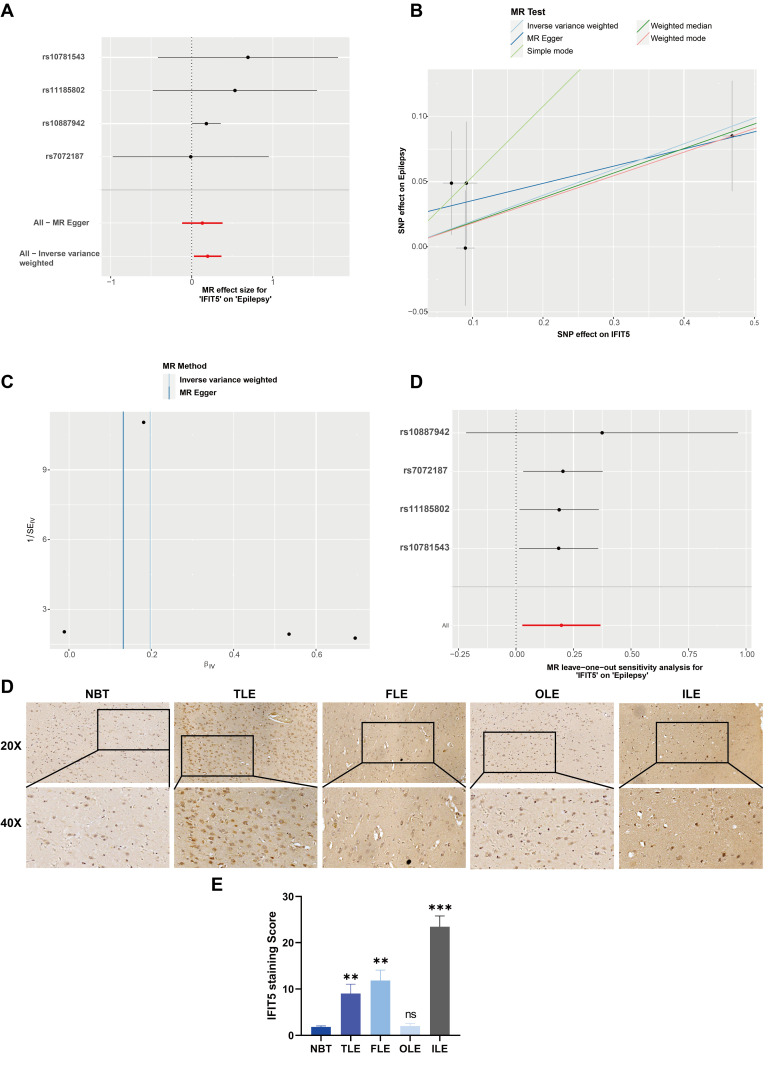
Mendelian randomization to identify differentially expressed m7G regulator causally links to epilepsy. (A) A forest plot showing the causal relationship between each SNP and the risk of epilepsy. (B) A scatterplot demonstrating how IFIT5 raises the likelihood of epilepsy. (C) A funnel plot shows the overall variability of the MR assessments of the effect of IFIT5 on epilepsy. (D) A leave-one-out figure showing how IFIT5 and the risk of epilepsy are related causally.

After rigorous screening to eliminate all weak instrumental variables, we comprehensively incorporated all SNPs satisfying the requirements into analysis. This investigation delved deeply into the intricate relationship between the mRNA expression levels of m7G-related genes and the predisposition to epilepsy, ultimately pinpointing genes that are causally implicated in the pathogenesis of this disorder. Notably, IFIT5 emerged as a significant factor, exhibiting an odds ratio (OR) greater than 1. This finding aligns with previous research that documented the upregulation of IFIT5 in epilepsy (Figs 7A, 7B, and 1A). Specifically, utilizing the inverse-variance weighted (IVW) approach, we calculated an OR of 1.218, with a 95% confidence interval (CI) ranging from 1.028 to 1.444, and a statistical significance of p = 0.023. The symmetry observed in the Funnel plot’s causal impact analysis suggests that there are no significant biases in the causal effects examined (Fig 7C), thereby bolstering the robustness of our findings. Subsequently, we systematically eliminated each SNP and repeated the Mendelian randomization (MR) analysis on the remaining SNPs. The consistency of our results underscores the robust causal link between the outcomes of all SNP computations (Fig 7D). Immunohistochemistry results confirmed that IFIT5 expression was broadly upregulated in the majority of primary epilepsy patient tissue samples, predominantly in neurons. However, in samples from patients with occipital lobe epilepsy (OLE), the upregulation of IFIT5 was not as pronounced compared to normal brain tissue. This suggests that IFIT5 and its regulatory m7G modification are generally upregulated in epilepsy patients (Fig 7E, F). Collectively, these findings point to a potential association between the upregulation of IFIT5 and the incidence of epilepsy, providing valuable insights into the underlying mechanisms of this complex neurological disorder.

### Comparing the significance of m6A and m7G in the context of epilepsy

In our prior investigation, we delved into the intricate biological mechanisms in which m6A may contribute to epilepsy, encompassing diverse aspects such as cellular demise modalities, glycometabolic pathways, and pharmacological responsiveness [14]. Nevertheless, the relative significance of m6A versus m7G, both being integral components of RNA editing, in the context of epilepsy remains elusive. To address this gap, we estimated the GSVA scores for m6A and m7G in each sample, revealing a noteworthy pattern: m6A exhibited an inhibitory trend while m7G displayed an activating profile in epilepsy patients (S7A and S7C Figs). The discriminatory power of these markers was further quantified by AUC values of 0.63 and 0.61, respectively (S7B and S7D Figs). Subsequently, we plotted the reverse cumulative distribution and boxplots of the absolute residuals (|residual|) to gain deeper insights (S7E and S7F Figs). Notably, the analysis of ROC curves underscored the robustness of the GLM model in this context (S7G Fig). To further strengthen our understanding, we calculated and compared the dropout losses resulting from permutations ranging from 0 to 10 for both m6A and m7G processes. Our findings indicate that the m7G GSVA score exhibited a lower dropout loss compared to the m6A GSVA score in epilepsy, suggesting, from a machine learning perspective, that the m7G process might hold a slightly more significant role in epilepsy patients than m6A (S7H–K Figs). Nonetheless, we interpret these results with caution, as they stem from preliminary transcriptomic analyses. Both m6A and m7G modifications are embedded within intricate, interconnected regulatory networks, each modulating distinct biological processes. In this context, the true “importance” of a pathway should be gauged by its potential to offer the greatest clinical benefit to patients.

## Discussion

A common neurological condition affecting over 50 million individuals worldwide is epilepsy. Recurrent occurrences might have a major negative impact on the patient’s quality of life [17]. It was also a risk factor for other neurological conditions including depression, Parkinson’s disease, and Alzheimer’s disease, since it was caused by aberrant neuronal firing [18–20]. Though the genetic mechanism behind it was still mostly unknown, several researchers noted that genetic influences were more significant than previously believed [21]. Furthermore, a recent analysis indicated that RNA editing should be investigated further because it was found to be closely linked to a number of neurological conditions, including epilepsy [22]. Thus, we looked at how m7G regulators work in epilepsy and what important pathways they could control.

After doing the differences analysis, we identified eight important m7G regulators in total. Then, we discovered that epilepsy sufferers’ expression of these genes differed from that of normal people. METTL1, NUDT3, EIF4E3, LARP1, EIF4A1, IFIT5, LSM1, and SNUPN were the eight regulators. The WD repeat domain 4 (WDR4) complex and methyltransferase-like 1 (METTL1) complex either enhanced or impeded the processes of several malignancies, including squamous cell carcinoma and lung cancer [23,24]. These studies suggest that METTL1 and m7G alteration may be used as biomarkers or as possible targets for interventions, opening up new avenues for the early detection and treatment of malignancies [25]. The part played by this gene in epilepsy has not yet been thoroughly investigated. Studies on Nudix (nucleoside diphosphate linked moiety X)-type motif 3 (Nudt3), an mRNA decamping enzyme that coordinates mRNA expression to control cell migration, provide more evidence that numerous decamping enzymes are active [26]. EIF4E3 and EIF4A1 are two examples of the coordinated eukaryotic initiation factors (eIFs) that regulate translation during initiation [27]. The eIF4E family consists of three members; eIF4E3, the third, is thought to bind m7G-cap in an unusual way and have tumor-suppressive properties in cells [28]. EIF4A1 was confirmed related to wound healing in cancers [29–31]. The cytological connection between eIF4E3, EIF4A1, and the beginning of epilepsy still need more research. The protein known as La-related protein 1 (LARP1) interacts to the terminal oligopyrimidine tract and the m7Gppp-C cap [32]. LARP1 and LARP4 work together to protect mRNA poly(A) through different molecular, targeting, and regulatory processes [33]. It has been discovered that interferon-induced protein with tetratricopeptide repeats (IFIT) proteins are crucial for controlling immunological responses, and that IFIT5 may help control traditional NF-κB activation [34]. Additionally, IFIT5 may affect the course of cancer and the epithelial-mesenchymal transition [35]. A part of the Lsm1–7 complex, LSM1 is responsible for the degradation of mRNA in the cytoplasm [36], which, according to studies on neurodevelopmental delay, is being studied as a potential novel candidate gene for neurodevelopmental disorders [37]. Besides, LSM1 is related to immune cell infiltration in tumor discovery [38]. Thus, we planned to investigate LSM1’s functions in the development of epilepsy, particularly immunological epilepsy. Snurportin 1 (SNUPN) was a snRNP-specific nuclear import receptor that selectively encoded proteins with m3G-cap. According to a recent study, SNUPN controlled the development of the cortical and lumbar regions and was linked to autism [39]. Regretfully, SNUPN’s research on neurological disorders is insufficient and requires more investigation.

Our research also revealed a strong relationship between m7G regulators and immune cell infiltration and functions, which may alter the immune microenvironment of neurons, impair normal function, and occasionally cause autoimmune epilepsy [40]. Microglia, as an often infiltrated immune cell, is widely considered to activate itself during epilepsy [41]. According to our findings, m7G activity was downregulated in epilepsy and greater in the mononuclear macrophage system than in other cells. Therefore, it makes sense to believe that the self-activation of these cells is connected to the downregulation of m7G levels in these cells. Owing to the unique ability of microglia to exhibit both neurotoxic and neuroprotective properties, it is challenging to determine the precise impact of this activation on the surrounding microenvironment [42,43]. Nonetheless, we are aware that m7G does encourage CD8 + T cell infiltration in tissue resolution since many methods indicated a favorable relationship between their numbers and m7G scores. They may induce seizures and harm the brain [44]. Besides, Alison Galloway et al. found that m7G cap methyltransferase (RNMT) is a key mediator of T cell activation, and specifically regulates ribosome production, which is consistant with our results [45]. Furthermore, we discovered that m7G was associated with the immune microenvironment and participated in several immunological processes, including cytolytic capacity. These results may serve as a foundation for further research on immunological systems regulated by m7G.

The primary mechanism that yields ATP, which is necessary for neuron development, signal processing, and regeneration, is oxidative phosphorylation [46,47]. However, neurons have unique anatomical characteristics that make it extremely difficult for them to maintain energy homeostasis [48]. When the oxidative phosphorylation mechanism is damaged, ROS and apoptotic factors may be released, which can lead to neuronal death [49]. We examined the expression conditions of genes connected to oxidative phosphorylation between m7G different patterns, taking into account these earlier investigations and the GSEA result’s proposal. Remarkably, the majority of DEGs in this pathway showed an improvement in their energy supply situation as seen by their up-regulation in the m7G-active pattern. A recent study related to oral squamous cell carcinoma pointed out that, as the main m7G writer, METTL1 could stimulate oxidative phosphorylation through m7G tRNA modification causally [50]. In short, the results indicate that m7G RNA editing could regulate the expression of oxidative phosphorylation associated genes, and promote this biological process in a few kinds of tissues.

Numerous neurodegenerative illnesses, including Alzheimer’s and Parkinson’s, can be brought on by neuron loss, a common pathological alteration associated with epilepsy [51–53]. It’s still unclear, though, if these neuronal fatalities are related to programmed cell death. The majority of the 12 types of programmed cell death, particularly necroptosis, were found to be downregulated in the m7G active pattern when we examined their differences. It is noteworthy that these findings were observed in neurons, aligning with a prior investigation that illustrated m7G modification in tRNA may enhance the proliferation of neuroblastoma cells derived from neurons and reduce their susceptibility to programmed cell death [54]. Additionally, analogous outcomes have been discovered in the context of acute myeloid leukemia, further validating the significance of these discoveries [55]. The findings suggested that the presence of m7G in epileptic patients’ brain neurons may be associated with minimizing neuronal damage and averting their programmed death, which is most likely mediated by enhancing energy supply and lowering ROS-induced neuronal damage.

## Conclusion

In this work, we screened five key m7G regulators in epilepsy and created a nomogram based on them to predict the risk of epilepsy, including METTL1, NUDT3, EIF4E3, LSM1, and SNUPN. Upon examining the many biological roles of m7G in epilepsy, we discovered that it has the ability to modulate the brain microenvironment of individuals with epilepsy, mostly manifested through chemotaxis and infiltration of CD8^+^ T cells. Furthermore, we discovered that m7G activity in neurons may stimulate oxidative phosphorylation, which may enhance the brain’s energy supply in epilepsy patients and lessen the disorder’s tendency to cause neurons’ programmed death, especially necroptosis. It is thought that this work will be able to direct future investigations into the molecular mechanism and genesis of epilepsy.

## Materials and methods

### Ethics statement

The Scientific Research Ethics Committee of Qilu Hospital, Shandong University examined and approved the study that used human subjects (approval number: KYLL-2022(ZM)-1303). And the patients or participants had provided their written informed consent to participate in this study. All methods were carried out in accordance with relevant guidelines and regulations.

### Data acquisition

We obtained RNA expression data from a total of 91 epilepsy patients and 51 normal persons in Gene Expression Omnibus (GEO) (https://www.ncbi.nlm.nih.gov/geo/) for subsequent research [56]. GSE143272 was the dataset used in detail. These people’s whole blood was used to extract the RNA expression data, which were then further examined using bioinformatics techniques. Finally, 19 m7G genes were retrieved in order to investigate the roles and potential pathways that m7G regulates in epilepsy research. Additionally, clinical characteristics and medical histories were gathered for a correlation analysis with m7G regulators in this dataset. Furthermore, the GSE190452 dataset, which includes brain tissue from four donors with temporal lobe epilepsy (TLE) and four donors without TLE, provides the single-cell sequencing data. In GEO, all of the data was freely accessible.

### Processing of single-cell sequencing data

R (version 4.3.0) included the “Seurat” package, which was primarily utilized for single-cell sequencing analysis [57]. Only those cells with percent.mt < 0.1 and nFeature_RNA > 50 were chosen. Ultimately, 22343 cells were chosen. Before starting the analysis, we performed inter-sample correction using the “RunHarmony” function in the “harmony” package (version 1.0.3) and normalization using the “NormalizeData” function [58]. In addition, we computed the top 2000 genes with the greatest expression differences among cells using the “FindVariableFeatures” method. For tSNE dimension reduction, the top thirty major components were chosen, and the resolution was set to 1. The “AddModuleScore” function was utilized to determine the scores for each pathway.

### Drawing of receiver operating characteristic curves

ROC curves were drawn using the “pROC” package in R (version 1.18.4) [59]. Besides, the best cut-off values [14] with sensitivity and specificity of each genes were calculated with R package “reportROC” (version 3.6).

### Consensus clustering based on significant m7G regulators

Consensus clustering was used to discover unique m7G patterns employing important m7G regulators. The “ConsensusClusterPlus” tool in R software (version 1.64.0) was utilized for this purpose. A technique for determining the number of unsupervised classes in a dataset that produced both quantitative and visual stability evidence was consensus clustering [60]. By dividing the epilepsy patients into a few subgroups based on important m7G regulators, we may speculate about the functions these regulators may play by an analysis of their differences.

### Principal component analysis (PCA) and calculation of m7G scores

To decrease the dimension, principal component analysis (PCA) was applied. PCA was widely used for high-dimensional data analysis and genome-wide expression investigations [61]. The “prcomp” function was employed in this study’s analysis. The formula to determine M7G Score is as follows: M7G Score = PC1 + PC2. In this stage, Z-scores were computed before analysis.

### Gene set variation analysis (GSVA) and gene set enrichment analysis (GSEA)

We examined the activity of all KEGG pathways in epilepsy patients using the “GSVA” package (version 1.48.3) in R. The results showed a good correlation between the total expression of each pathway’s genes and the activity of the patients’ KEGG pathways [62]. Version 4.2.3 of the “GSEA” software is used to implement GSEA. Enrichment scores (ES) were computed using weighted statistics akin to Kolmogorov-Smirnov. The likelihood that a route was thought to be abundant in it increased with its ES [63]. We examined the variations in the KEGG pathway across various m7G clusters of epileptic patients using these two techniques.

### Microenvironment analysis of epilepsy patients

IOBR is a simple method for using multi-omics data to advance immuno-oncology research [64]. We used the “ESTIMATE” method with this instrument to calculate the stromal and immunological scores of epilepsy patients [65]. The relative abundance of the various cell types is determined using the TIMER2 website (http://timer.cistrome.org/) and single sample gene set enrichment analysis (ssGSEA). TIMER2 is an online program that includes seven immune microenvironment prediction algorithms, such as “TIMER” and “CIBERSORT” [66]. In addition, ssGSEA was carried out using the “GAVA” package in R (version 1.48.3) [62].

### Identification of differentially expressed genes (DEGs) between distinct m7G patterns

Differentially expressed genes (DEGs) between m7G clusters A and B were acquired and screened using the “limma” package in R (version 3.56.2), which stands for “Linear Models for Microarray Data” and is available as a R package [67]. The cutoff conditions were set at |log2FC| > 0.5 and FDR < 0.05.

### Construction of prediction model based on multiple machine learning algorithms

To quantify the relevance of eight key m7G regulators, we used multiple machine learning methods with the “caret” R package (version 6.0-94), such as the random forest model (RF), generalized linear model (GLM), and support vector machine model (SVM). First, we calculated and showed each model’s residual using the “Reverse cumulative distribution of residual” and “Boxplots of residual” functions of the R package “DALEX”. Furthermore, R software’s “pROC” package (version 1.18.4) was used to quantify their effectiveness [59]. Finally, we chose SVM for further statistical studies. It allows users to create a hyperplane in the characteristic space with a maximum margin to differentiate between positive and negative examples [68]. The five most important m7G regulators were then identified.

### Construction of the nomogram model

To predict the probability of epilepsy, a nomogram model was created using the R package “rms” (version 6.7−1). The calibration curve was plotted to ensure that the projected and reality values were consistent. A decision curve analysis (DCA) was used to see whether this model could benefit epilepsy patients. We also conducted a clinical effect analysis to assess its importance [69].

### Calculation of cell death scores

Based on the “GSVA” package in R (version 1.48.3), the cell death scores of bulk RNA sequencing data were calculated using the ssGSEA algorithm [62].

### RNA extraction and RT-qPCR

The total cell RNA was extracted from the whole blood and brain tissue of epilepsy patients and non-epilepsy individuals using TRIzol, as directed by the manufacturer. Next, we reversed the transcription using High Capacity cDNA Reverse Transcription Kits (Applied Biosystems). After extracting the cDNA, real-time PCR was performed using the quantitative PCR System Mx-3000P (Stratagene). The detailed PCR primer sequences are presented in S2 Table.

### Calculation of m6A and m7G GSVA Scores

The GSVA Scores of m6A and m7G were calculated by R package “GSVA” (version 1.48.3) [62].

### Mendalian randomization

Utilizing the R package “TwoSampleMR” (version 0.5.10), we rigorously defined single-nucleotide polymorphisms (SNPs) as instrumental variables (IVs) and implemented a double-sample Mendelian randomization (MR) approach to elucidate the potential causal relationship between the RNA expression of specific genes and the risk of developing epilepsy [70]. The datasets employed in this comprehensive study were sourced from the IEU OpenGWAS project (https://gwas.mrcieu.ac.uk/), specifically the datasets with identifiers “ finn-b-GE_STRICT”. Prior to analysis, SNPs with an F-statistic value less than 10, indicative of weak instrumental strength, were excluded to ensure the robustness of our findings. We further employed inverse-variance weighted (IVW) regression to quantitatively assess the association between gene expression levels and disease risk. Additionally, MR-Egger regression was utilized in subsequent sensitivity analyses to strengthen the credibility of our conclusions.

### Immunohistochemistry

Paraffin-embedded brain tissue samples from different regions of epileptogenic foci were sectioned at a thickness of 4 µm and mounted on microscope slides. The sections were deparaffinized in xylene and rehydrated through a graded ethanol series to water. Antigen retrieval was performed by heating the sections in 10 mmol/L citrate buffer (pH 7.2) in a microwave oven. Sections were then incubated overnight at 4°C with the primary antibody IFIT5 (1:200, Protentech, 13378–1-AP). After rinsing with PBS, the sections were incubated with horseradish peroxidase (HRP)-conjugated goat anti-rabbit secondary antibody (ZSGB-BIO, PV-9000) for 30 minutes at room temperature. Visualization was achieved using diaminobenzidine (DAB) substrate (ZSGB-BIO, ZLI-9033). The sections were counterstained with Mayer’s hematoxylin (Beyotime Biotechnology, C0107), dehydrated through a graded series of ethanol, cleared in xylene, and mounted with a coverslip.

### Statistical analysis

Spearman linear regression analyses were used to determine the correlation between two continuous variables. Differences between two and numerous sets of data were found using the “Wilcoxon” and “Kruskal-Wallis” tests, respectively. Furthermore, p-value < 0.05 was regarded statistically significant. All statistical analyses were conducted using R software (version 4.2.3) and Graphpad Prism (version 9.5.1).

## Supporting information

S1 Fig Preprocessing of Single Cell Sequencing Data (A) Effect and quality of sequencing shown by vlnplots. (B) Correlation between nCount RNA and percent mt or nFeature RNA. (C) PCA plot and vlnplot of embeddings value before harmonization. (D) PCA plot and vlnplot of embeddings value after harmonization. (E) Brain cells used were divided into 36 clusters shown by tSNE plot. (F) Expression of conventional markers in each type of cells.
(PNG)

S2 Fig Consensus clustering of epilepsy patients based on DEGs between m7G clusters. (A) Consensus matrix of patients when k = 2. (B) Boxplot of m7G score of patients between gene clusters. (C) Heatmap showing expression of DEGs between gene clusters. (D) Differentially expression of 8 significant m7G regulators between gene clusters displayed by boxplot. *p < 0.05, **p < 0.01, ***p < 0.001.
(PNG)

S3 Fig Analyses of Immune microenvironment and functions between gene clusters (A) Correlation between significant m7G regulators and immune cell infiltration. (B) Differences of immune functions between gene clusters. (C) Differences of immune cell infiltration between gene clusters. *p < 0.05, **p < 0.01, ***p < 0.001.
(PNG)

S4 Fig Relationship between m7G regulators and clinical factors (A) Clinical heat map including expression of m7G regulators of epilepsy patients. (B-E) Relationship between m7G score and age (B), gender (C), subtype of epilepsy (D), and drug-response (E). (F) Sankey diagram displaying relationship among three grouping methods.
(PNG)

S5 Fig M7G regulators may improve oxidative phosphorylation in brain cells of epilepsy. (A) Differentially expressed genes related to oxidative phosphorylation between gene clusters. (B) M7G scores of TLE patients’ brain cells, TLE1 with higher scores than others. (C-H) Expression of oxidative phosphorylation related genes in each TLE patients’ brain cells, including 6 parts, which are NADH Dehydrogenase (C), Cytochrome C Reductase (D), Cytochrome C Oxidase (E), Succinate Dehydrogenase (F), Cytochrome C (G), and V-type ATPase (H). *p < 0.05, **p < 0.01, ***p < 0.001.
(PNG)

S6 Fig Differences of other programmed cell death between high- and low- m7G TLE patients. Alkaliptosis (A), apoptosis (B), cuproptosis (C), entotic cell death (D), lysosome dependent cell death (E), netotic cell death (F), oxeiptosis (G), and parthanatos (H) are mostly different activated between high- and low-m7G TLE, with all brain cells included.
(PNG)

S7 Fig Slightly greater significance of m7G over m6A in epilepsy via multiple machine learning algorithms. Comparison of m6A (A) and m7G (C) GSVA Scores in normal subjects and epileptic patients. ROC analysis of m6A (B) and m7G (D) GSVA Scores in epilepsy. Reverse cumulative distribution (E) and boxplot (F) of absolute residuals (|residual|) for m6A and m7G. ROC curves (G) of three machine learning algorithms. (H-K) Lower dropout loss values for m7G compared to m6A across all three algorithms (H), GLM (I), SVM (J), and RF (K). *p < 0.05, **p < 0.01, ***p < 0.001.
(PNG)

S1 Table Baseline characters of epilepsy patients between distinct m7G clusters. (XLSX)

S2 Table Detailed sequences of primers for PCR. (XLSX)

## Abbreviations

RT-qPCR: Real Time Quantitative PCR
WHO: World Health Organization
m6A: N6-methyladenosine
PCA: Principal component analysis
tSNE: t-Distributed Stochastic Neighbor Embedding
GSVA: Gene Set Variation Analysis
ssGSEA: single sample Gene Set Enrichment Analysis
KEGG: Kyoto Encyclopedia of Genes and Genomes
ES: enrichment scores
RF: random forest
GLM: generalized linear model
SVM: support vector machine
ROC: Receiver OperatingCharacteristic
DCA: Decision curve analysis
AUC: Area Under Curve
eIFs: eukaryotic initiation factors
CNS: Central Nervous System
ROS: reactive oxygen speciesC
