# Supplementary figures and images for: Integrative Role of RNA N7-methylguanosine in epilepsy: Regulation of neuronal oxidative phosphorylation, programmed death and immune microenvironment
Source: PLoS One. 2025 Jul 14;20(7):e0327256. doi: 10.1371/journal.pone.0327256 (PMC12258582; doi:10.1371/journal.pone.0327256)

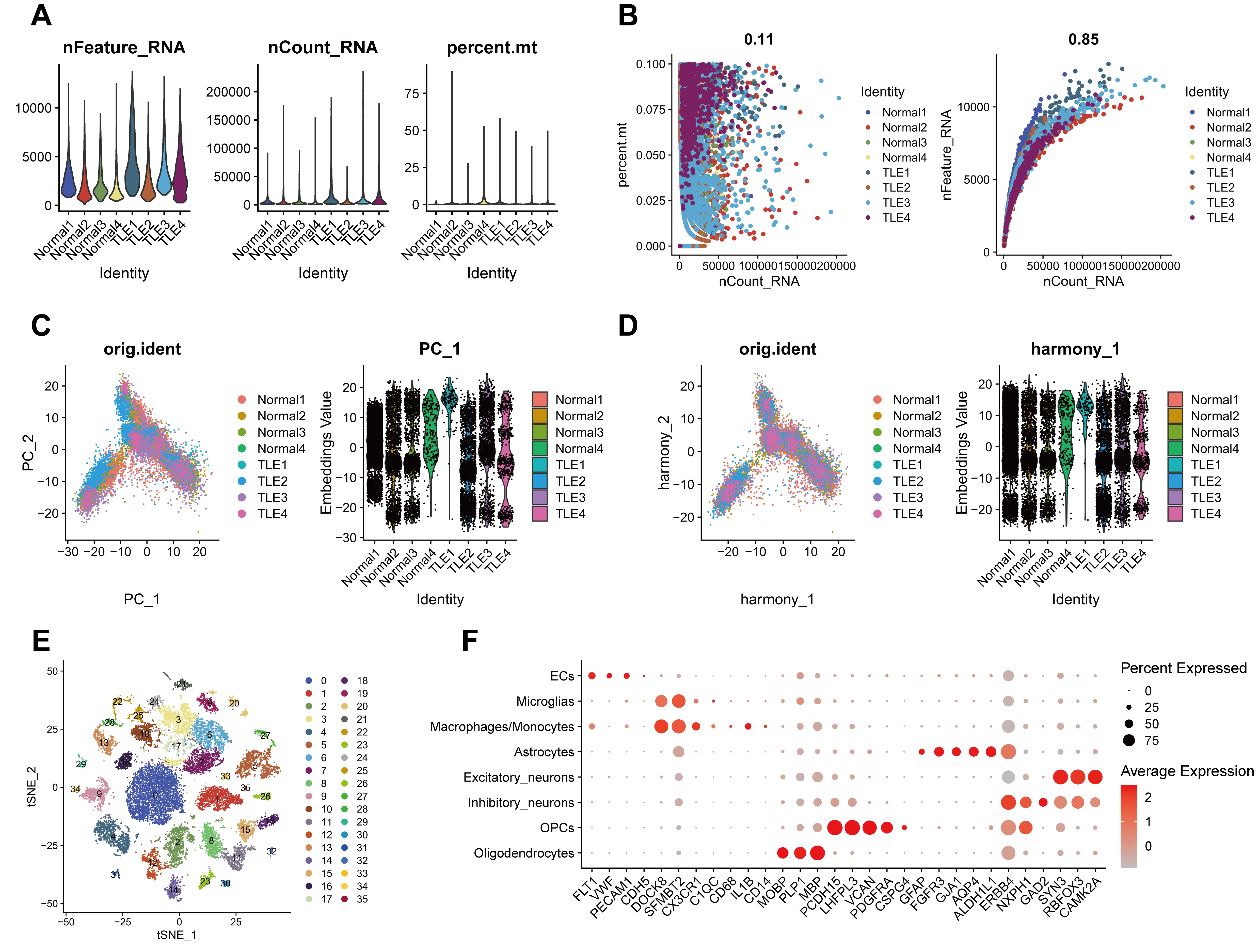

Supplement: S1 Fig — (A) Effect and quality of sequencing shown by vlnplots. (B) Correlation between nCount RNA and percent mt or nFeature RNA. (C) PCA plot and vlnplot of embeddings value before harmonization. (D) PCA plot and vlnplot of embeddings value after harmonization. (E) Brain cells used were divided into 36 clusters shown by tSNE plot. (F) Expression of conventional markers in each type of cells. (PNG) [file pone.0327256.s001.png]

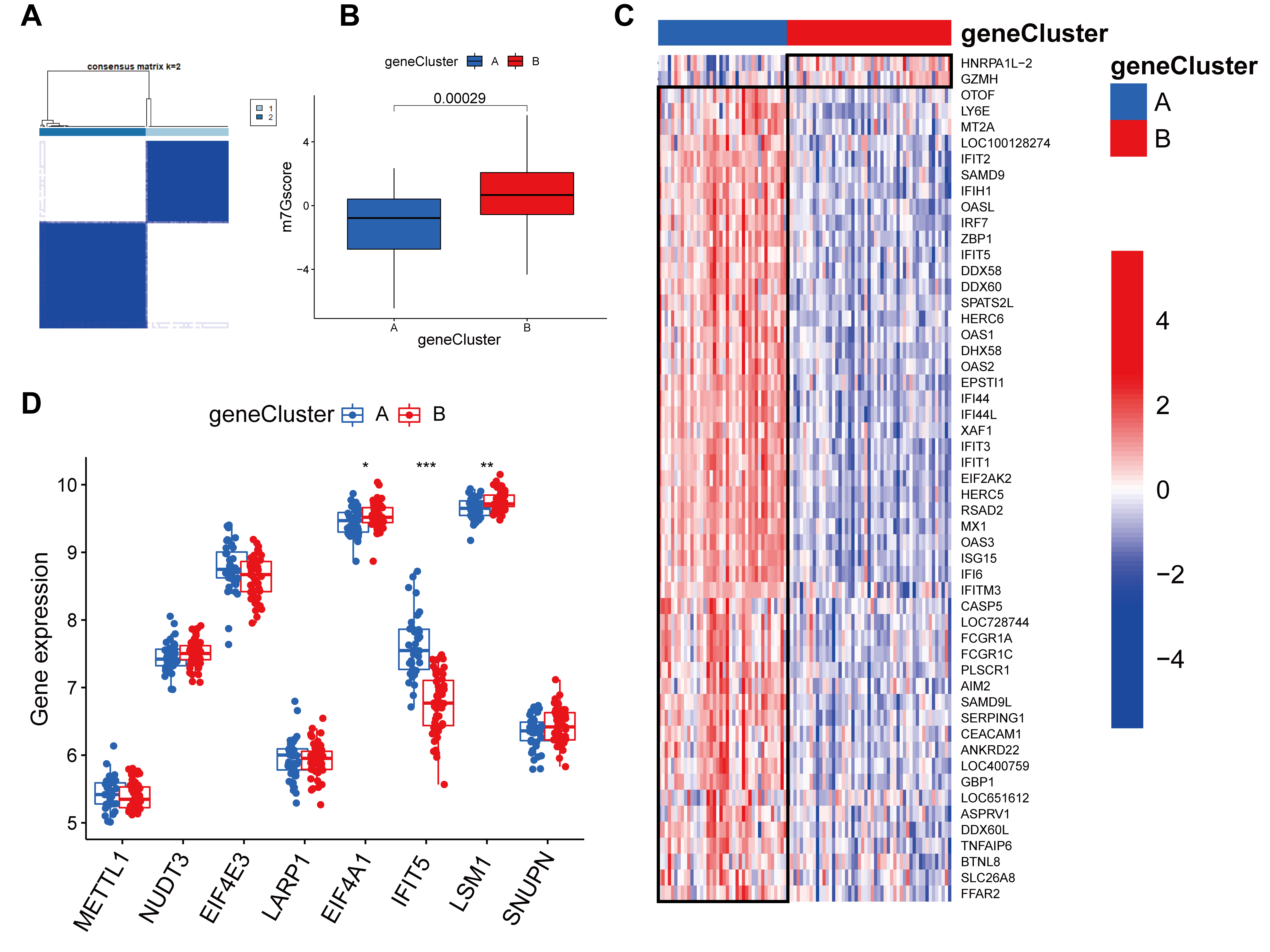

Supplement: S2 Fig — (A) Consensus matrix of patients when k = 2. (B) Boxplot of m7G score of patients between gene clusters. (C) Heatmap showing expression of DEGs between gene clusters. (D) Differentially expression of 8 significant m7G regulators between gene clusters displayed by boxplot. *p < 0.05, **p < 0.01, ***p < 0.001. (PNG) [file pone.0327256.s002.png]

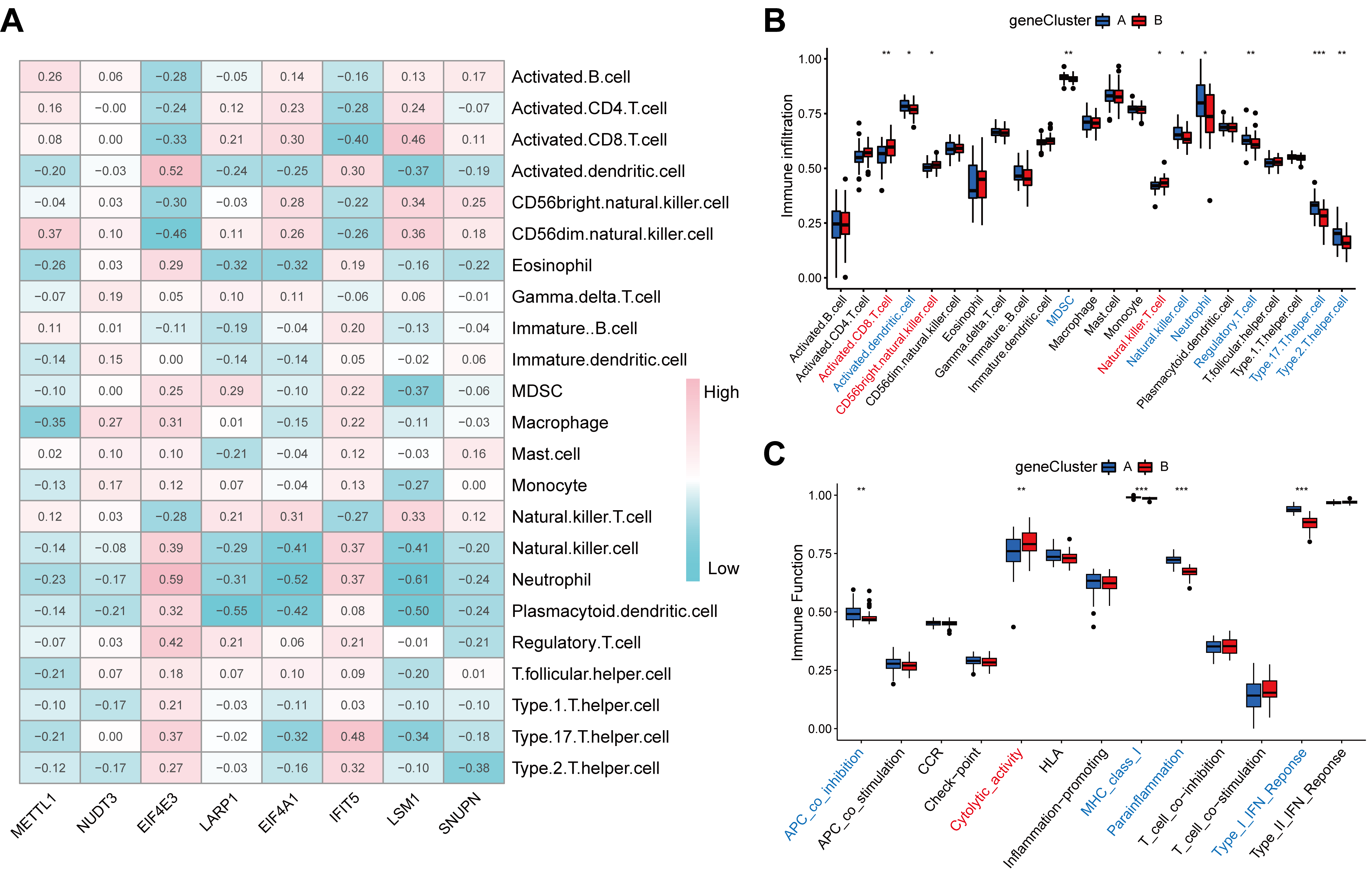

Supplement: S3 Fig — (A) Correlation between significant m7G regulators and immune cell infiltration. (B) Differences of immune functions between gene clusters. (C) Differences of immune cell infiltration between gene clusters. *p < 0.05, **p < 0.01, ***p < 0.001. (PNG) [file pone.0327256.s003.png]

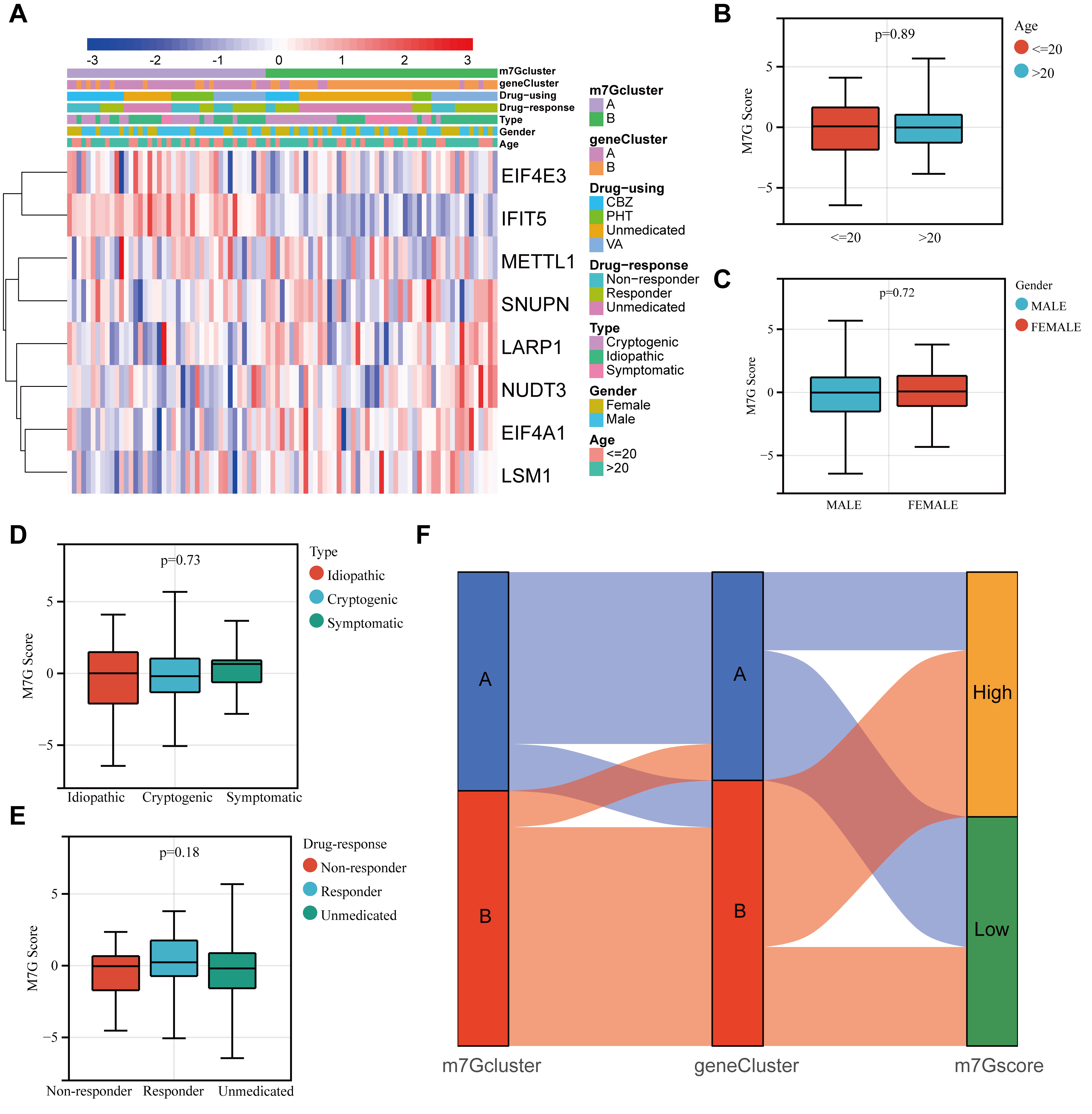

Supplement: S4 Fig — (A) Clinical heat map including expression of m7G regulators of epilepsy patients. (B-E) Relationship between m7G score and age (B), gender (C), subtype of epilepsy (D), and drug-response (E). (F) Sankey diagram displaying relationship among three grouping methods. (PNG) [file pone.0327256.s004.png]

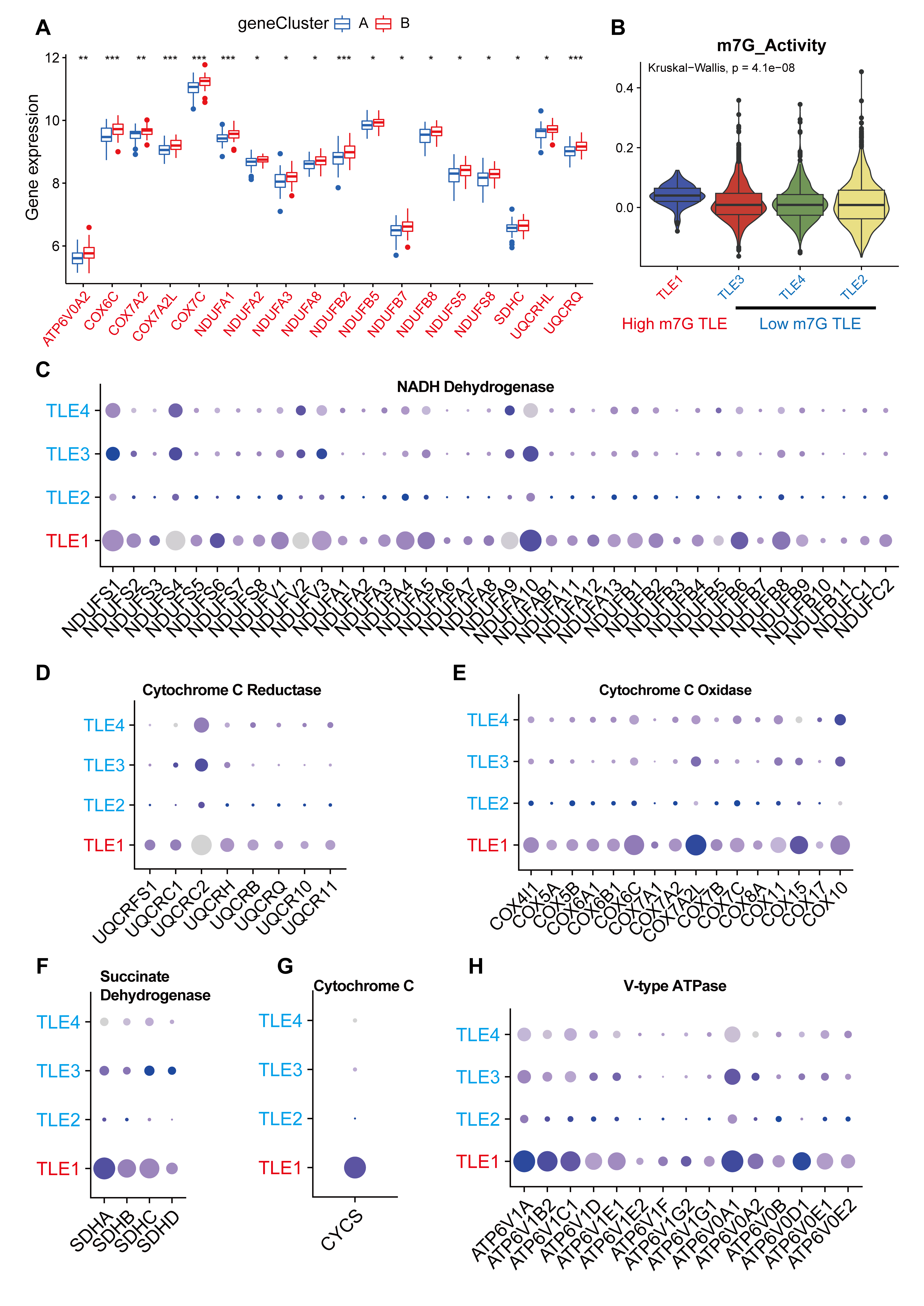

Supplement: S5 Fig — (A) Differentially expressed genes related to oxidative phosphorylation between gene clusters. (B) M7G scores of TLE patients’ brain cells, TLE1 with higher scores than others. (C-H) Expression of oxidative phosphorylation related genes in each TLE patients’ brain cells, including 6 parts, which are NADH Dehydrogenase (C), Cytochrome C Reductase (D), Cytochrome C Oxidase (E), Succinate Dehydrogenase (F), Cytochrome C (G), and V-type ATPase (H). *p < 0.05, **p < 0.01, ***p < 0.001. (PNG) [file pone.0327256.s005.png]

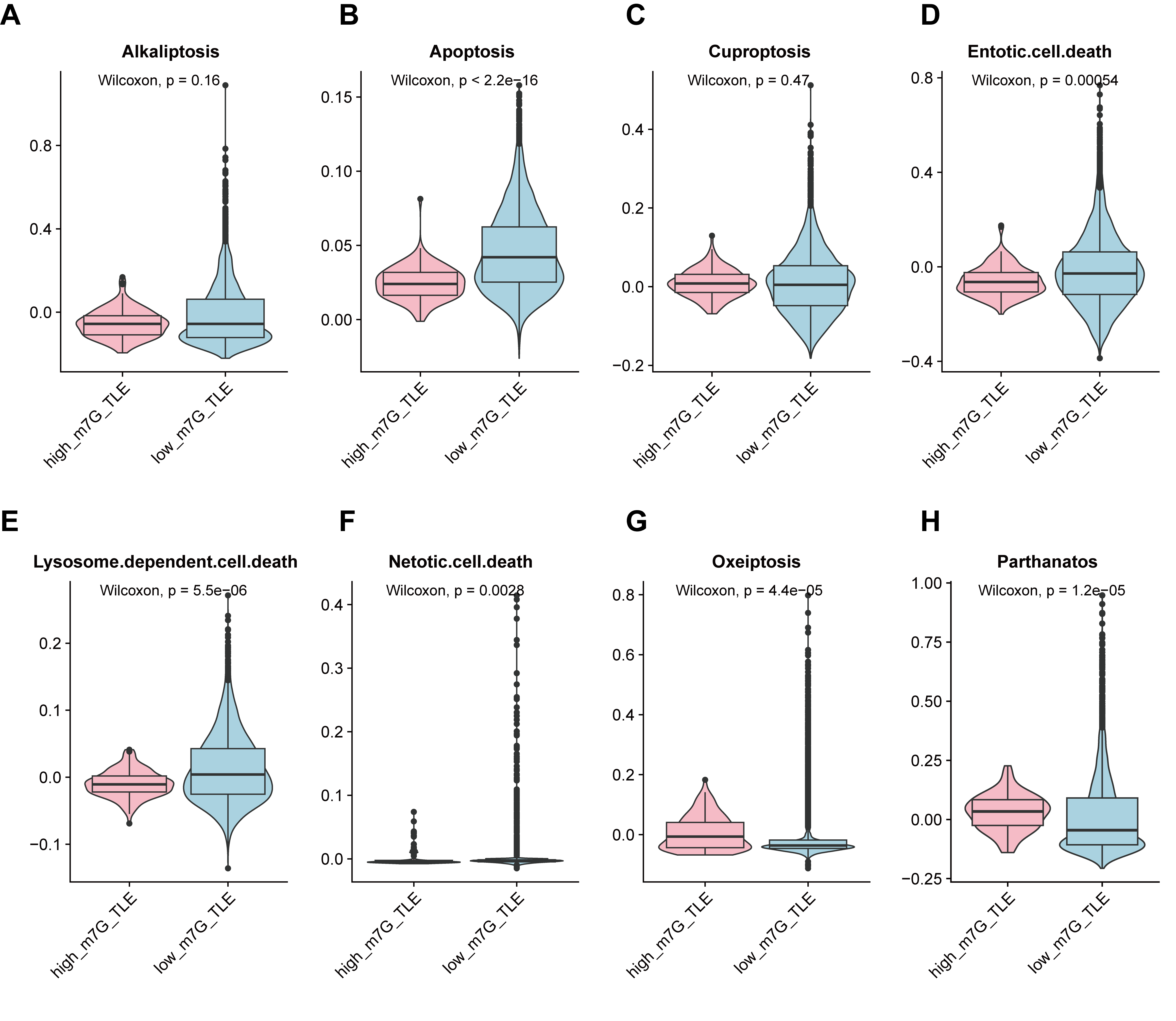

Supplement: S6 Fig — Alkaliptosis (A), apoptosis (B), cuproptosis (C), entotic cell death (D), lysosome dependent cell death (E), netotic cell death (F), oxeiptosis (G), and parthanatos (H) are mostly different activated between high- and low-m7G TLE, with all brain cells included. (PNG) [file pone.0327256.s006.png]

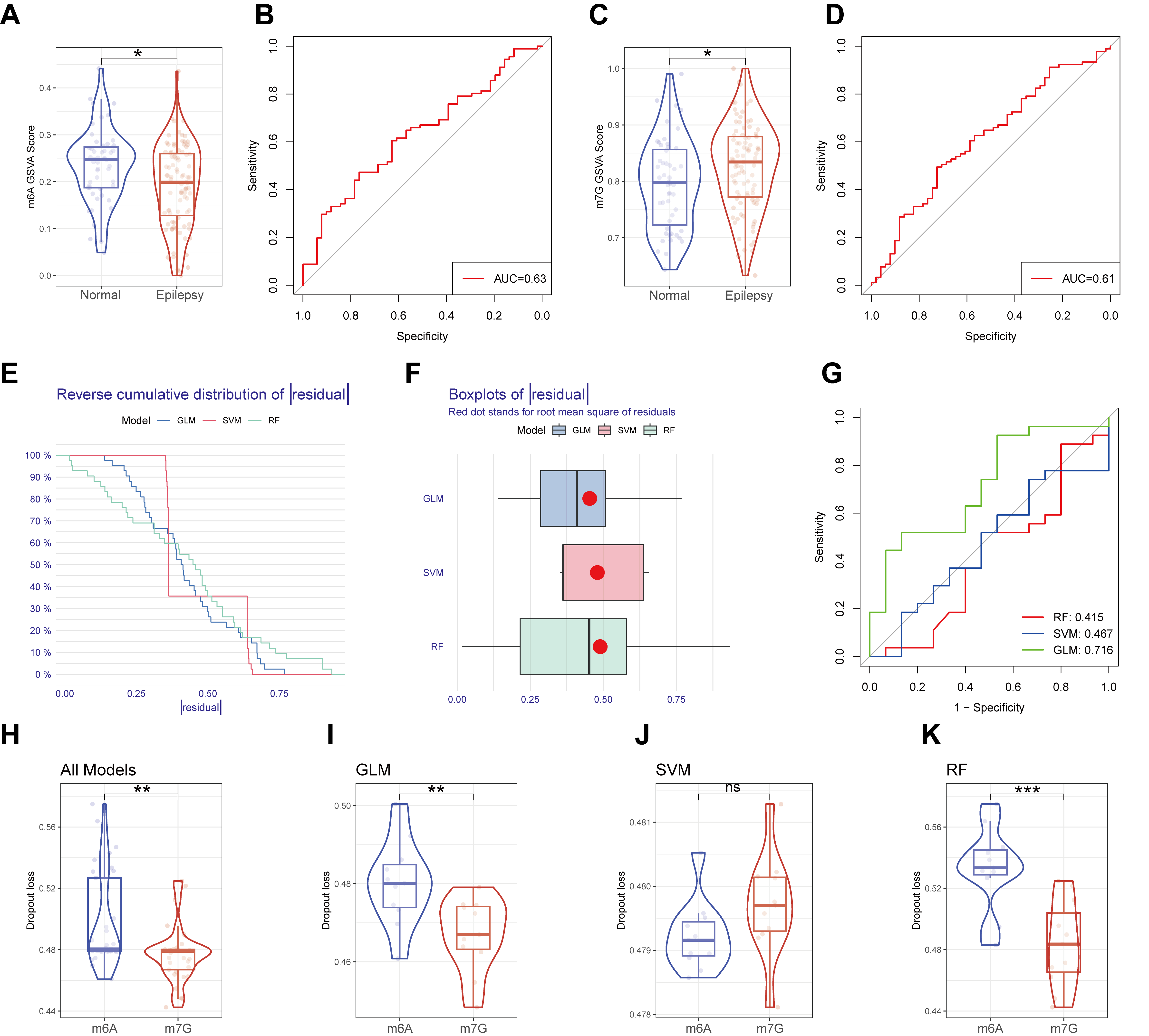

Supplement: S7 Fig — Comparison of m6A (A) and m7G (C) GSVA Scores in normal subjects and epileptic patients. ROC analysis of m6A (B) and m7G (D) GSVA Scores in epilepsy. Reverse cumulative distribution (E) and boxplot (F) of absolute residuals (|residual|) for m6A and m7G. ROC curves (G) of three machine learning algorithms. (H-K) Lower dropout loss values for m7G compared to m6A across all three algorithms (H), GLM (I), SVM (J), and RF (K). *p < 0.05, **p < 0.01, ***p < 0.001. (PNG) [file pone.0327256.s007.png]
